# Earn Your Own Living

**Published:** 1873-01

**Authors:** 


### EARN YOUR OWN LIVING.

Let every parent teach his child that it is more manly and more honorable to earn his own living than to be helped to it by inheritance; that there is something small and mean in leaning upon another; that it is the height of nobleness to depend on one's own strong arm. To this end begin early, begin before the infant can turn over in its cradle; lay it on the floor and place tempting things within sight, to be had only by crawling to them; let the general idea be that a child is not to be helped by another to do what it can do itself.

Any spook may make money if a fortune is put into his hands with which to operate, but he is the true man who makes his own capital to begin with; these are they who, in the near future, will be the Astors, and Coopers, and Stewarts, and Vanderbilts of their day and generation.

A young Dutchman was one day trudging along Broadway with a heavy pack of fur on his back, his wife following after who had a genius for determining the quality and value of any skin, domestic or wild; the man was John Jacob Astor, and the woman behind him helped to make his fortune.

Peter Cooper worked a year for twenty-five dollars, and has given a million since to aid and encourage young boys and young girls to get into the way of earning a living for themselves.

A. T. Stewart began life with a clerical education and a thousand dollars, now the owner of millions, giving several of these millions to afford a respectable and plentiful and tidy home, at the cost of three or four dollars a week (room, fuel, gas, washing and eating included), but only for women who are dependent on their own work for a living.

Vanderbilt earned his first dollar by rowing persons across the Hudson for twelve and a half cents each; to-day his death would be considered a great calamity to the city and State of New York, it would be a nation's regret. It is not known that Mr. Vanderbilt gives much money to help the poor and the striving; but he has given a grand example to the young men of his time. His philosophy of success is, "Do your work well, and keep down expenses," and if any considerable number of the young will but learn the lesson and are stimulated by his example to "hoe their own row" in life he will not have lived in vain.

A large part of the charities of our time are worse than thrown away. No human being ought to be supported in idleness who is not physically or mentally incapable of doing anything toward his own support; with this exception, every man or woman who is helped by others should be made to help themselves as far as possible. The happiest road in life is that which is traveled in earning one's own living.

